# Retrospective cohort study monitoring PEG-asparaginase activity in acute lymphoblastic leukemia patients with and without premedication

**DOI:** 10.12688/f1000research.19298.2

**Published:** 2020-01-17

**Authors:** Michael Losasso, Bruce Bostrom, Yoav Messinger

**Affiliations:** 1 University of St. Thomas, 2115 Summit Avenue, St. Paul, Minnesota, 55105, USA; 2 Cancer and Blood Disorders, Children's Minnesota, 2525 Chicago Avenue South, Minneapolis, Minnesota, 55404, USA

**Keywords:** Pegasparginase Activity, Premedication, Silent Inactivation, Allergy, Anaphylaxis

## Abstract

**Background: **PEG-L-asparaginase (pegaspargase) is a critical component of therapy for children and adults with acute lymphoblastic leukemia (ALL). Allergic reactions, which may occur in up to one third of patients, are the major cause for discontinuation. One study reported lower rates of allergic reactions with premedication. Besides allergy, an unknown number of patients develop silent neutralizing antibodies not associated with allergic reactions. The purpose of this retrospective cohort study was to determine the incidence of silent inactivation of pegasparaginase and compare incidence of allergic reactions with and without premedication.

**Methods: **Using a commercial assay, asparaginase activity was monitored following pegaspargase (2500 units/m ) in newly diagnosed children and young adults with B- and T-cell ALL from February 2013 to May 2017. The incidence of allergic reactions before and after initiation of premedication in May 2015 was compared.

**Results: **One patient out of 59 (1.7%) had silent inactivation after the second dose. No patient had silent inactivation after the first pegaspargase dose and no standard risk B-cell ALL patients, who received only two pegaspargase doses in combination with oral dexamethasone, had silent inactivation. The incidence of grade 3 or 4 allergic reactions was 3.7% per dose with premedication (methylprednisolone, acetaminophen and diphenhydramine) versus 5.2% without. The incidence per patient with premedication given for most of the doses was 8.3% versus 17% without. These values are not statistically significant. Premedication did not affect pegaspargase activity.

**Conclusions: **Due to the low incidence of silent inactivation with intravenous pegaspargase and the unlikely event patients receiving only two doses of pegasparaginase would receive erwinase for this possible transient silent inactivation, we recommend routine monitoring of pegaspargase activity only in patients scheduled to receive more than two doses.

## Introduction

PEG-L-asparaginase (pegaspargase) is a critical component of therapy for children and adults with acute lymphoblastic leukemia (ALL). Its use is hampered by many issues including allergic reactions, silent inactivation, thrombosis, hyperbilirubinemia and pancreatitis ^1^. Other common toxicities, such as hyperglycemia and hypertriglyceridemia, may be mitigated with the use of metformin ^2^ and omega-3 ^3^ and fenofibrate ^4^. There is also ongoing interest in the use of carnitine to treat, and possibly prevent, hepatic toxicity, manifested by a severe increase in direct bilirubin, among other findings ^5^.

The optimal dose, dose interval and target asparaginase level for pegaspargase is not completely established ^6^. In pediatrics, a dose of 2500 units/m ^2^ is the norm, whereas for adult patients, doses are often reduced due to increased toxicity at the pediatric dose. Some investigators have suggested using a pharmacokinetic driven model to individualize pegaspargase dosing ^7^. 

The use of premedication (acetaminophen, diphenhydramine and a corticosteroid) has been suggested as a possible means of reducing allergic reactions. In a multi-center study testing the use of pediatric-based regimens in young adults, the rate of grade 3 or 4 allergic reactions was reduced from 10% to 4% after premedication was mandated ^8^. A study in adults with ALL reported allergic reactions in 7.2% of patients when pegaspargase was given concurrently with, or followed by, one week of prednisone ^9^. Using a novel mouse model of asparaginase hypersensitivity, pretreatment with seven days of oral dexamethasone was the only agent capable of mitigating the severity of hypersensitivity and partially restoring asparaginase activity ^10^. Dexamethasone given at the time of, or for one week following, asparaginase was not as effective.

The presence of antibodies against asparaginase may be found, from as early as the end of induction therapy ^11, 12^. The presence of asparaginase antibodies had sensitivity of 87–88% and specificity of 68–69% for clinical reactions ^11^. The presence of asparaginase antibodies at end of induction did not appear to alter prognosis in a large multi-center study ^12^. This suggests that measuring asparaginase activity is more useful than looking for the presence of antibodies.

Silent inactivation of pegaspargase activity by anti-asparaginase antibodies or other immune-mediated mechanisms are potentially of greater concern than bone-fide allergic reactions, as patients with grade 3–4 allergic reactions to pegaspargase will be switched to erwinase, which theoretically will improve outcome. The true incidence of silent inactivation is unknown, as there are no reports of a comprehensive screening program for silent inactivation in a large multi-institutional trial. The largest published study found silent inactivation in 7/89 (8%) of patients ^13^. However, these patients received induction with native *Escherichia coli* asparaginase before switching to pegaspargase, which is not current practice. The authors also report in the same group of patients that silent antibodies may spontaneously resolve with continued pegaspargase ^14^. Notably, lower silent inactivation with pegaspargase than native *Escherichia coli* asparaginase have been reported ^15– 17^ Prudence suggests that patients who receive premedications should have pegaspargase activity monitored after every dose, due to the possible but unproven concern that premedication will mask allergic reactions and silent inactivation. In fact, a consensus panel of experts recommends screening for silent inactivation in all patients undergoing therapy for ALL with asparaginase ^18^. Additionally, the low grade 1–2 allergic reactions that are more common than silent inactivation do require pegaspargase activity monitoring to ensure a switch to erwinase if confirmed as true inactivation.

## Methods

### Ethical statement

As the use of premedications and measurement of pegaspargase activity was considered by the leukemia provider group at Children’s Minnesota to be necessary for optimal care, no informed consent was obtained. Parents/adult patients were not informed of results unless intervention was indicated, which did not occur. This retrospective review study was approved by the institutional review board of Children’s Minnesota (IRB# 1606-062).

### Patients

This retrospective study occurred in a large pediatric oncology center that diagnoses and treats approximately 40 new cases of ALL yearly in children and young adults up to age 30. If there are open studies, the patients are enrolled on Children’s Oncology Group protocols. Otherwise, patients are treated according to the most recent risk adapted protocols for standard risk B, high risk B and T-ALL. In order to reduce acquisition bias, charts of every patient in first remission who received pegaspargase from December 2013 to September 2016 were abstracted (N=99). As this was a pilot study and the expected reduction of grade 3 or 4 allergic reactions with premedications was unknown at the time, sample sizes calculations could not be calculated. Data from all 99 patients were used to estimate the incidence of grade 3 or 4 allergic reactions by patient and by dose. For the detailed pharmacokinetic analysis, we used a subgroup of all patients from May 2014 to September 2016 (N=46) who had pegaspargase levels drawn. This number was sufficient to define the confidence intervals of the pegaspargase activity.

### Pegaspargase administration

A total of 112 blood samples from these 46 patients were collected from a central venous portacath in conjunction with scheduled clinical visits from 3 to 12 days following pegaspargase administration at the standard dose of 2500 mg/m ^2^. Pegaspargase was given by intramuscular injection or intravenously per Children’s Oncology Group protocols on an intermittent schedule starting with induction and completed prior to starting maintenance therapy. Because the distribution of the collection days clustered in ranges from day 3–5, 6–8 and 10–12, for analyses, pegaspargase activity was grouped in these categories. One data point was omitted from analysis in this version of the manuscript because it was and extreme outlier that was not congruent with other values from the patient or group (UPN 11; day 7 after 3 ^rd^ dose; value 2.86; greater than 99.9 ^th^ percentile). Two data points were removed because they were drawn after anaphylaxis and as expected undetectable (UPN 38 and 42 after 2 ^nd^ dose). These values have been retained in the online dataset. To better estimate the incidence of silent inactivation, pegaspargase levels lower than 0.01 units/ml were looked for in the data from an additional 13 patients making a total of 59 evaluated. No evidence of silent inactivation was found in these 13 patients.

These patients were all treated according to Children’s Oncology Group protocols, using either intramuscular or intravenous pegaspargase as the only form of asparaginase. Intramuscular asparaginase was the standard of care until 2010 when intravenous administration became the new standard of care based on the Children’s Oncology Group AALL0932 protocol ^19^. A comprehensive review of published studies concluded that the risk of grade 3 or 4 allergic reactions is independent of the pegaspargase route of administration ^19^.

### Premedication administration

We became aware of an abstract showing a decrease in grade 3 or 4 allergic reactions in a multi-institutional study employing pegaspargase in young adults with ALL ^20^. This prompted us to institute in May 2015 strict manditory premedication with acetaminophen (10–15 mg/kg orally), diphenhydramine (1 mg/kg orally or intravenously), and methylprednisolone (1 mg/kg intravenously), within the hour prior to administering pegaspargase. Every subsequent patient was to receive with all three of the premedication drugs without exception. The numbers with and without premedication are listed in Table 2 (per pegaspargase dose) and Table 3 (per patient).

### Assessment of allergic reactions

Allergic reactions to were graded per CTC 4.0 toxicity scales. We compared the incidence of grade 3 or 4 allergic reaction in patients with and without premedication, both per pegaspargase dose and per patient. 

### Pharmacokinetic analysis

Routine monitoring of pegaspargase activity in patients with ALL was initiated in 2013 after the ‘asparaginase activity analysis’ test approved by Clinical Laboratory Improvement Amendments was introduced by AIBioTech, Richmond, VA 23225 US. Subsequent to the introduction in 2015 of a quantitatively identical test by Next Molecular Analytics, Chester, VA, samples were exclusively sent there.

### Statistical analyses

SPSS version 23 was used for graphing and analyses. Grouped data were displayed with box graphs depicting the ~1 ^st^, 25 ^th^, 50 ^th^ (median), 75 ^th^ and ~99 ^th^ percentiles. The data was normally distributed by visual inspection of the normal curve and Kolmogorov-Smirnov test, with the exception of the day 6–8 pegaspargase activity level. Removal of one extreme outlier as described above rendered the day 6–8 pegaspargase activity level normally distributed. The comparison of pegaspargase activity with and without premedication was done by independent sample t-test. The comparison of grade 3 or 4 allergic reactions by patient and pegaspargase dose with and without the use of premedication was done by chi-squared analysis. As some premedication doses were missed due to omission by the treating physician, an additional analysis of the incidences of those who received premedication after every dose or most doses were compared to those who received no premedication before any dose. Missed pegaspargase activity samples were omitted from analysis ( Table I).

**Table 1.** Percent of pegaspargase activity specimens collected following doses one to nine.

	One	Two	Three	Four	Five	Six	Seven	Eight	Nine	Total
Collected	8	33	18	19	18	6	7	2	1	112
Missed	38	12	16	10	7	6	3	3	1	96
Total	46	45	34	29	25	12	10	5	2	208
% Collected	17%	73%	53%	66%	72%	50%	70%	40%	50%	54%

**Table 2.** Grade 3–4 allergic reactions by use of premedication per dose of pegaspargase.

Premeds used with the dose	Total doses	Doses with allergic reaction	Percent of doses with grade 3–4 allergic reaction
no	155	8	5.2%
yes	185	7	3.7%

**Table 3.** Grade 3–4 allergic reactions by use of premedication per patient.

	Grade 3–4 allergic reactions	None	Total
No premedication	7 (17%)	35 (83%)	42
Some premedication	4 (6%)	58 (94%)	62
Every dose premedication	4 (12%)	30 (88%)	34

## Results

Pharmacokinetic analyses were done on 100 specimens from 46 patients. The 46 patients included 12 standard risk B-cell patients, 21 high risk B-cell ALL patients, and 13 T-cell ALL patients. There were 25 males and 21 females. The ages ranged from one to 29 years with a median of 8.3 years. The number of specimens and missed specimens per pegaspargase dose number are shown in Table 1. These include all patients who had pegaspargase activity collected which included those eliminated from other analyses as they were not in first remission. First dose specimens were frequently missed, whereas specimens on doses two to seven were collected at least half the time.

**Figure 1.**
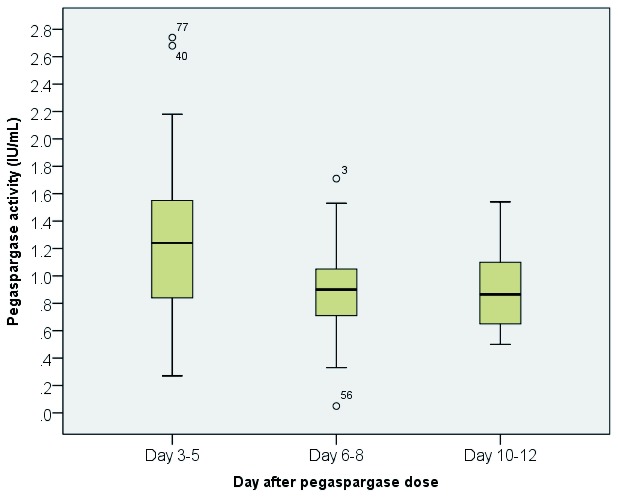
Box plot of pegaspargase activity following 2500 units/m ^2^ on day 3–5, 6–8 and 10–12. The data point below the line is from the patient with silent inactivation. Data points outside of the whiskers of the ~1 ^st^ and ~99 ^th^ percentiles are represented by a circle (outlier more than 1.5 times the interquartile range). The attached number is a data point and not a value.

Figure 1 is a box and whisker graph of pegaspargase activity on days 3–5, 6–8 and 10–12. The mean, standard error of the mean, standard deviation and number of data points are: 1.37, 0.21 0.76 units/mL and 13, respectively, for day 3–5; 0.88, 0.04, 0.31 units/mL and 61 for day 6–8; 0.89, 0.06, 0.28 units/mL and 26 for day 10–12. These values are similar to those previously reported in pediatric patients with ALL ^21, 22^.

**Figure 2.**
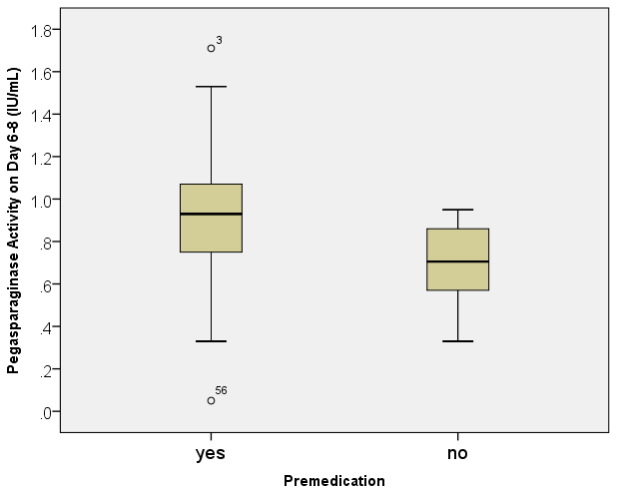
Box plot of pegaspargase activity day 6–8 after 2500 units/m ^2 ^with and without premedication. Patients who received premedication has a significantly greater value (p=0.003). The mean and standard deviation for the premedication group is 0.93 and 0.32 units/mL and for the no premedication group is 0.69 and 0.21 units/mL. Data points outside of the whiskers of the ~1 ^st^ and ~99 ^th^ percentiles are represented by a circle (outlier more than 1.5 times the interquartile range). The attached number is a data point and not a value.

Figure 2 is box and whisker graph of the pegaspargase activity on day 6–8, following doses with or without premedication. This time ranged was used for the comparison as it is the most common time for checking asparaginase activity. The mean and standard deviation for the no premedication group is 0.69 and 0.21 units/mL (N=12 samples), respectively, and for the premedication group is 0.93 and 0.32 units/mL (N=49 samples). These were significantly different by independent samples t-tests for equal variances not assumed (p = 0.003). The day 3–5 data had only one patient in the no premedication group so was not analyzed. The day 10–12 data showed higher median value in the group that received premedications (0.92 vs 0.83) which was not statistically significant due to insufficient power.

Only one patient had silent inactivation with the following activity levels by dose number and day following pegaspargase activity was checked: dose 1, day 24 - 0.11 units/mL; dose 2, day 8 - 0.05 units/mL; dose 3 day 6 - 0.01 units/mL; dose 4 day 8 - 0.33 units/mL; dose 5, day 8 - 0.82 units/mL; and dose 6, day 10 - 0.62 units/mL The low values after doses 2 and 3 were not reviewed due to a clerical error until after dose 4, which showed adequate activity, so pegaspargase was continued until the end of treatment. 

For the analysis of the role of premedication in preventing grade 3–4 allergic reactions, the data was analyzed per pegaspargase dose and per patient. In the analysis per pegaspargase dose, premedication did not significantly reduce grade 3–4 allergic reactions. However due to insufficient numbers the power was only 0.24. With premedication, 7/185 (3.7%) had grade 3–4 allergic reactions compared to 8/155 (5.2%) without premedication ( Table 2).

Table 3 shows the incidence of grade 3–4 allergic reactions per patient. Without premedication, 7/42 (17%) had grade 3–4 allergic reactions. When premedication was given most of the time (usually the first dose was missed), 4/62 (6%) had grade 3–4 allergic reactions. When premedication was given for every dose, 4/34 (12%) had allergic reactions. There was no significant effect of premedication on grade 3–4 allergic reactions by dose when the premedication group (8/96; 8.3%) was compared to the no premedication group (7/42; 17%) (chi square = 2.09; p = 0.15) ( Table 3). However due to insufficient numbers the power was only 0.54. There was no difference in the distribution of patients who did or did not receive premedication by risk group.

## Discussion

Compared with historical controls that received similar therapy, premedication did reduce the incidence of grade 3 or 4 allergic reactions when measured per patient or per dose of pegaspargase. The power calculation was only 0.54 per patient and 0.24 per pegaspargase dose, thus our study was underpowered to show statistical significance due to insufficient numbers. As premedication does not negatively affect pegaspargase activity levels, and other studies using historical comparisons have suggested premedication may reduce allergic reactions, we are continuing the practice ^8, 9^.

The interesting observation by Tong *et al.* that asparaginase antibodies generated after native *E. coli* asparaginase may resolve while on pegaspargase continuation therapy needs to be confirmed in patients who receive only pegaspargase during induction and beyond ^14, 19^. We noted a transient decrease in pegaspargase activity, likely due to silent inactivating antibodies in 1/59 patients (1.7%). This decrease of pegaspargase activity occurred after the second dose in a high-risk B-cell ALL patient and resolved with continuation of pegaspargase dosing. No decrease in pegaspargase activity was seen in standard risk patients who received only two doses of pegaspargase in combination with oral dexamethasone. This observation is limited by small numbers of patients and multiple missed levels after the first pegaspargase dose.

Limitations of the study include multiple missed activity levels that may have found additional patients with silent inactivation. The sample size also makes it difficult to estimate the true incidence of silent inactivation and if premedication reduces the incidence of grade 3 or 4 allergic reactions. Additional studies are needed to clarify this. Another limitation is the patients had only one activity level per pegaspargase dose, limiting evaluation of true pharmacokinetics especially in patients with higher or lower than expected levels.

 We found low incidence of silent inactivation with intravenous pegaspargase. Our study suggests that patients treated with regimens that include only two doses of pegaspargase, given with dexamethasone, may not need asparaginase levels due to even lower silent inactivation, but this would need confirmation by larger studies. We do suggest that patients who have questionable allergic reactions (grade 1–2) would benefit from asparaginase levels that will direct switch to erwinase if confirmed as true inactivation.

Our data showed a statistically significantly greater pegaspargase activity of the day 6–8 pegaspargase level with the use of premedication. Analysis of the day 3–5 and 10–12 pegaspargase levels did not show a significant difference likely due to the small sample sizes.

The finding of difference in the pegaspargase level with premedication has not been previously described to our knowledge. This may be of clinical significance and should, if possible confirmed in other retrospective studies. Given the increased acceptance of premedication for pegaspargase it is unlikely and perhaps unethical to confirm in a prospective randomized trial, in our opinion. 

### Addendum

A recent publications by 2 other groups also reported reduction of infusion reactions and need for erwinase using premedication. They also found a low incidence of silent inactivation with intravenous pegaspargase of 1.5% in one report and < 1% in another report similar to our finding of one in 59 patients (1.7%) ^23, 24^.

## Data availability

### Underlying data

Underlying data available at https://doi.org/10.6084/m9.figshare.8281826.v1
