# Supplementary material for: Retrospective cohort study monitoring PEG-asparaginase activity in acute lymphoblastic leukemia patients with and without premedication
Source: F1000Res. 2020 Jan 17;8:1007. Originally published 2019 Jul 4. [Version 2] doi: 10.12688/f1000research.19298.2 (PMC7001754; doi:10.12688/f1000research.19298.2)
Supplement: Peer review history

# Peer review: Reviewer response for version 2

**DOI:** 10.5256/f1000research.24084.r58717

**Published:** 2020-02-04

**Authors:** David J. Young

**Affiliations:** 1 Translational Stem Cell Biology Branch of the National Heart, Lung, and Blood Institute, National Institutes of Health (NIH), Bethesda, MD, USA

The authors have responded to each of the comments by all three reviewers, making the suggested corrections, providing acceptable alternative approaches, or explaining why the changes would not be possible. These revisions have not raised any new concerns.

I confirm that I have read this submission and believe that I have an appropriate level of expertise to confirm that it is of an acceptable scientific standard.

# Peer review: Reviewer response for version 1

**DOI:** 10.5256/f1000research.21156.r53445

**Published:** 2019-10-01

**Authors:** David J. Young

**Affiliations:** 1 Translational Stem Cell Biology Branch of the National Heart, Lung, and Blood Institute, National Institutes of Health (NIH), Bethesda, MD, USA

This report by Losasso, Bostrom and Messinger expand upon our growing understanding of the appropriate treatment with and management of asparaginase-based therapies in lymphoblastic leukemia. In this retrospective study, the authors have reviewed the single center experience of therapeutic drug monitoring, examining patient drug levels to determine the incidence of drug inactivation and looked at the relationship between pre-treatment and serious adverse events.

 In their review, the authors found a 1.7% rate of drug inactivation. Of note, this rate is comparable to several different prior studies which the authors have referred here. Although the authors did not measure directly for inactivating antibodies, the use of drug activity levels to impute the presence of antibodies is now considered an acceptable approach as it is generally the primary determinant of drug activity for most patients, and as such is not a limitation in this work.

 Their observation of transient inactivation and then recovery of drug efficacy is an important finding, and bears future examination as this strongly argues, as others have, for rechallenging patients with prior history, especially in high-risk patients where receiving multiple doses of asparaginase has been conclusively linked to better outcomes.

 Contrary to other studies, the authors remark that they did not observe a significant decrease in clinically significant (grade 3-4) reactions with predication. Although statistically correct, it is difficult to support this statement for several reasons. First, although the authors do not see a ***statistically*** significant effect, there is without a doubt a ***clinically important*** trend of 50% reduction in events. Indeed, this would represent an absolute risk reduction of 8.7% with a number needed to treat of about 11, and is almost identical to other studies. *Post hoc* power analysis, although an admitted abuse of statistics, suggests that this data may have reached significance with 30-40 additional patients.

 Furthermore, the authors themselves continue to use and argue for premedication, which would argue that they agree that there is benefit to pre-medication despite the lack of statistical findings. Yet, the only reason for pre-medication, given the historic concerns regarding silent inactivation, is to reduce severe adverse reactions. These contradictions need to be resolved.

 Finally, the authors suggest that monitoring of drug levels is not necessary in low-risk patients, given that they did not identify inactivation during the first two doses. This statement is too strong. The authors themselves admit that there was a large number (83%) of missed levels during the first administration, likely a consequence of the admittedly chaotic logistics of early induction therapy experienced at many institutes. Although their data do suggest that silent inactivation  following one dose would likely still be detectable at the second dose, this has not been rigorously tested, and is based primarily upon the experience of a single patient. Therefore, to include the recommendation of not testing low-risk patients, especially its inclusion within the abstract that (regrettably) may be all that some read, and without mentioning the rate of missed levels in the same abstract, is difficult to support.

I confirm that I have read this submission and believe that I have an appropriate level of expertise to confirm that it is of an acceptable scientific standard, however I have significant reservations, as outlined above.

# Response

**Published:** 2019-12-19

**Authors:** Bruce Bostrom

**Affiliations:** Children's Hospitals and Clinics of Minnesota, Minneapolis, USA

David Young Reviewer 3

This report by Losasso, Bostrom and Messinger expand upon our growing understanding of the appropriate treatment with and management of asparaginase-based therapies in lymphoblastic leukemia. In this retrospective study, the authors have reviewed the single center experience of therapeutic drug monitoring, examining patient drug levels to determine the incidence of drug inactivation and looked at the relationship between pre-treatment and serious adverse events.

In their review, the authors found a 1.7% rate of drug inactivation. Of note, this rate is comparable to several different prior studies which the authors have referred here. Although the authors did not measure directly for inactivating antibodies, the use of drug activity levels to impute the presence of antibodies is now considered an acceptable approach as it is generally the primary determinant of drug activity for most patients, and as such is not a limitation in this work.

Their observation of transient inactivation and then recovery of drug efficacy is an important finding, and bears future examination as this strongly argues, as others have, for re-challenging patients with prior history, especially in high-risk patients where receiving multiple doses of asparaginase has been conclusively linked to better outcomes.

Contrary to other studies, the authors remark that they did not observe a significant decrease in clinically significant (grade 3-4) reactions with predication. Although statistically correct, it is difficult to support this statement for several reasons. First, although the authors do not see a ***statistically*** significant effect, there is without a doubt a ***clinically important*** trend of 50% reduction in events. Indeed, this would represent an absolute risk reduction of 8.7% with a number needed to treat of about 11 and is almost identical to other studies. *Post hoc* power analysis, although an admitted abuse of statistics, suggests that this data may have reached significance with 30-40 additional patients.

Furthermore, the authors themselves continue to use and argue for premedication, which would argue that they agree that there is benefit to pre-medication despite the lack of statistical findings. Yet, the only reason for pre-medication, given the historic concerns regarding silent inactivation, is to reduce severe adverse reactions. These contradictions need to be resolved.

Response: we agree with the reviewer points and his astute observations. We would have included his *Post hoc* analysis if we could but were concerned like him about those who would view these statistics unkindly. Several things were altered: In Discussion 1 ^st^ paragraph this sentence is now changed: “Compared with historical controls that received similar therapy, premedication did reduce the incidence of grade 3 or 4 allergic reactions when measured per patient or per dose of pegaspargase. The power calculation was only 0.54 per patient and 0.24 per pegasparaginase dose thus our study was underpowered to show statistical significance.” The 4 ^th^ paragraph this whole section was removed: “Contrary to the findings of a large multi-institutional trial, where the introduction of premedication significantly reduced the incidence of high-grade allergic reactions, our study did not show a statistically significant reduction with the use of premedication. [7] Despite this, we continue to use premedication in all patients receiving pegasparaginase.”

Finally, the authors suggest that monitoring of drug levels is not necessary in low-risk patients, given that they did not identify inactivation during the first two doses. This statement is too strong. The authors themselves admit that there was a large number (83%) of missed levels during the first administration, likely a consequence of the admittedly chaotic logistics of early induction therapy experienced at many institutes. Although their data do suggest that silent inactivation following one dose would likely still be detectable at the second dose, this has not been rigorously tested, and is based primarily upon the experience of a single patient. Therefore, to include the recommendation of not testing low-risk patients, especially its inclusion within the abstract that (regrettably) may be all that some read, and without mentioning the rate of missed levels in the same abstract, is difficult to support.

Response: we do agree with the reviewer completely that the number of missed levels after the 1 ^st^ dose is very high. We also agree that our numbers are too small for this strong conclusion. We have altered the abstract to include this sentence in the results: “Asparaginase dose levels were mostly missed after the first asparaginase dose.” We eliminated this sentence: “No patient had silent inactivation after the first pegaspargase dose.” And the conclusions: “We found low incidence of silent inactivation with intravenous pegaspargase. Future studies may confirm if patients treated with regimens with only two doses of pegasparaginase, given with dexamethasone, may not need asparaginase levels due to even lower silent inactivation.”

In discussion 2 ^nd^ paragraph this was altered: “No decrease in pegaspargase activity was seen in standard risk patients who received only two doses of pegaspargase in combination with oral dexamethasone. This observation is limited by small numbers of patients and multiple missed levels after the first pegaspargase dose.” The 4 ^th^ paragraph was altered also: “We found low incidence of silent inactivation with intravenous pegaspargase. Our study suggests that patients treated with regimens that include only two doses of pegasparaginase, given with dexamethasone, may not need asparaginase levels due to even lower silent inactivation, but this would need confirmation by larger studies.”

- **Is the work clearly and accurately presented and does it cite the current literature?**Yes
- **Is the study design appropriate and is the work technically sound?**Yes
- **Are sufficient details of methods and analysis provided to allow replication by others?**Yes
- **If applicable, is the statistical analysis and its interpretation appropriate?**Yes
- **Are all the source data underlying the results available to ensure full reproducibility?**Yes
- **Are the conclusions drawn adequately supported by the results?**Partly

Competing Interests *No competing interests were disclosed.*

# Peer review: Reviewer response for version 1

**DOI:** 10.5256/f1000research.21156.r53513

**Published:** 2019-09-30

**Authors:** Vinod Pullarkat

**Affiliations:** 1 Department of Hematology and Hematopoietic Cell Transplantation, Gehr Family Center for Leukemia Research, City of Hope Medical Center, Duarte, CA, USA

In this manuscript the authors have retrospectively examined the incidence of allergic reactions in a cohort of patients with ALL receiving multiple doses of PEGylated aspraginase. They show that premedication did not have an impact on allergic reactions. The study has drawbacks inherent in a retrospective analysis of a heterogeneously treated population with non standardized blood sampling as acknowledged by the authors. My comments are as follows:

1. A concern with premedication is whether it will mask a true antibody mediated allergy. A major question therefore is whether the allergic reactions observed were actually antibody mediated allergic reactions or infusion reactions that do not affect asparaginase activity. Therefore authors should show the asparaginase levels of patients who had a reaction and compare to those who did not, in order to show whether the reactions observed are due to drug neutralizing antibodies.
2. In their conclusion (in the abstract) the authors focus on patients who receive only 2 doses of PEG asparaginase. In most regimens patients will receive more doses of PEG asparaginase and hence it is unclear why authors focus on patients who receive only 2 doses. The correct conclusion in my opinion is that premedication did not impact the incidence of allergic reaction and premedication did not mask silent inactivation.

I confirm that I have read this submission and believe that I have an appropriate level of expertise to confirm that it is of an acceptable scientific standard, however I have significant reservations, as outlined above.

# Response

**Published:** 2019-12-19

**Authors:** Bruce Bostrom

**Affiliations:** Children's Hospitals and Clinics of Minnesota, Minneapolis, USA

**Vinod Pullarkat: **Department of Hematology and Hematopoietic Cell Transplantation, Gehr Family Center for Leukemia Research, City of Hope Medical Center, Duarte, CA, USA 

In this manuscript the authors have retrospectively examined the incidence of allergic reactions in a cohort of patients with ALL receiving multiple doses of PEGylated asparaginase. They show that premedication did not have an impact on allergic reactions. The study has drawbacks inherent in a retrospective analysis of a heterogeneously treated population with non-standardized blood sampling as acknowledged by the authors. My comments are as follows:

1. A concern with premedication is whether it will mask a true antibody mediated allergy. A major question therefore is whether the allergic reactions observed were actually antibody mediated allergic reactions or infusion reactions that do not affect asparaginase activity. Therefore authors should show the asparaginase levels of patients who had a reaction and compare to those who did not, in order to show whether the reactions observed are due to drug neutralizing antibodies.

Response: It would have been wonderful to be able to do this, but we cannot. Unfortunately, when patients had a reaction (be it allergic reaction or infusion reaction) no further pegaspargase was given. Therefore, these patients had no subsequent asparaginase activity measured. Additionally, this is an inherent difficulty with these patients – whose infusion is stopped right away and they never receive full dose of pegaspargase rendering subsequent asparaginase activity meaningless.

 

1. In their conclusion (in the abstract) the authors focus on patients who receive only 2 doses of PEG asparaginase. In most regimens patients will receive more doses of PEG asparaginase and hence it is unclear why authors focus on patients who receive only 2 doses. The correct conclusion in my opinion is that premedication did not impact the incidence of allergic reaction and premedication did not mask silent inactivation.

Response: We agree completely. The abstract and the paper has changes that completely address this. To the Abstract conclusion we added this sentence: “Though premedication resulted in lower allergic reactions the difference was not statistically different due to insufficient power.” In discussion the 1 ^st^ paragraph completely addresses this: “Compared with historical controls that received similar therapy, premedication did reduce the incidence of grade 3 or 4 allergic reactions when measured per patient or per dose of pegaspargase.  The power calculation was only 0.54 per patient and 0.24 per pegasparaginase dose, thus our study was underpowered to show statistical significance due to insufficient numbers.”

The comment suggesting not monitoring levels in patients receiving only two doses of pegasparaginase is to potentially minimize unnecessary testing in this group of patients.  In COG protocols only two doses of pegasparaginase are given to lower risk children who make up the majority of patients under the age of 18. However we have removed this recommendation due to large number of first dose pegaspargase levels missed in these patients.

- **Is the work clearly and accurately presented and does it cite the current literature?**Yes
- **Is the study design appropriate and is the work technically sound?**Yes
- **Are sufficient details of methods and analysis provided to allow replication by others?**Yes
- **If applicable, is the statistical analysis and its interpretation appropriate?**Partly
- **Are all the source data underlying the results available to ensure full reproducibility?**No
- **Are the conclusions drawn adequately supported by the results?**Partly

Competing Interests: *Speakers Bureau and Advisory board for Servier and Jazz pharmaceuticals*

Reviewer Expertise:Treatment of acute lymphoblastic leukemia

**I confirm that I have read this submission and believe that I have an appropriate level of expertise to confirm that it is of an acceptable scientific standard, however I have significant reservations, as outlined above.**

# Peer review: Reviewer response for version 1

**DOI:** 10.5256/f1000research.21156.r53512

**Published:** 2019-09-26

**Authors:** Bernard L. Marini

**Affiliations:** 1 Department of Pharmacy Services and Clinical Sciences, Michigan Medicine, University of Michigan College of Pharmacy, Ann Arbor, MI, USA

Drs. Losasso, Bostrom, and Messinger should be applauded for their work in characterizing asparaginase activity levels in ALL patients. The results and research are clearly presented and accurately reported. The conclusions of the study are appropriate, and the limitations of this retrospective cohort study are well-described.

 Some specific suggestions/comments/recommendations:

**Abstract:**

- I would include the n in the incidence of hypersensitivity reactions, or acknowledge that the study may not have been powered to assess this outcome. Readers who only look at the abstract may wrongly conclude pre-medication is not effective, when the analysis may have been underpowered to detect a statistically significant difference in hypersensitivity reactions in the pre-medication vs. no pre-medication group.
- The paper, methods, and title are focused on the impact of pre-medication on activity levels; however, the conclusion primarily talks about the low incidence of silent inactivation with two doses; thus monitoring only being necessary in patients that receive >2 doses. I would consider a sentence re: pre-medication here as well. 

**Intro:**

- Not essential, but could consider mentioning fibrates in the treatment of hypertriglyceridemia, which is another first line option.
- Would cite more recent data on the impact of pre-medication on preventing hypersensitivity reactions - Cooper SL, Young DJ, Bowen CJ, Arwood NM, Poggi SG, Brown PA. Universal premedication and therapeutic drug monitoring for asparaginase-based therapy prevents infusion-associated acute adverse events and drug substitutions. Pediatr Blood Cancer. 2019 Aug;66(8):e27797. ^1^ and Marini BL, Brown J, Benitez L, Walling E, Hutchinson RJ, Mody R, Jasty Rao R, Slagle L, Bishop L, Pettit K, Bixby DL, Burke PW, Perissinotti AJ. A single-center multidisciplinary approach to managing the global Erwinia asparaginase shortage. Leuk Lymphoma. 2019 May 17:1-15 ^2^.
- I would be careful with stating asparaginase antibodies are always predictive of future allergic reactions, as not all antibodies inactivate asparaginase or lead to clinical hypersensitivity reactions (ex: CCG01961). Consider rewording to soften this language.
- In paragraph 5, the statement is made that silent inactivation is of greater concern than allergic reactions. However, one could argue that allergic reactions are more common than silent inactivation with asparaginase (~1%), and monitoring activity levels in the setting of grade 1-2 allergic reactions is essential to determine which patients with clinical hypersensitivity reactions are having true antibody mediated reactions vs. other, non-antibody mediated (possibly ammonia vs. other immune-mediated) allergic reactions that would not warrant a switch to another asparaginase preparation.
- Other large scale studies assessing the rate of silent inactivation and finding the rate to be <8% could also be included in this discussion (or cited). These include, AALL07P4, Schore, et al. Leuk Lymph 2019 ^3^, CCG1962, and Park JH, et al. Blood. 2016;128:1629 ^4^.

**Methods:**

- While a sample size was not calculated a priori, could the authors conduct a sensitivity analysis on the data determined the detectable effect size at 80% power in the primary outcome of interest (rate of grade 3/4 allergic reactions?)?
- Were dose reductions in PEG ever conducted in this data set for toxicity/age/risk factors for hepatotoxicity?
- It is unclear how silent inactivation is defined in this data-set from the methods. It may also be clinically relevant to capture "accelerated clearance", which also may be clinically relevant. Not all anti-asparaginase antibodies lead to full inactivation of the drug, but some lead to accelerated clearance and lower than expected values at early time points. A patient with silent inactivation would have a peak level that is undetectable, whereas a patient with accelerated clearance may clear the PEG by day ~7 when an expected duration of asparagine depletion is 21-28 days with a full pediatric dose. 
- Were any levels obtained in patients with low-grade hypersensitivity reactions? If so, did any of these patients have accelerated clearance/"loud" inactivation?
- Were the activity levels normally distributed? If not, perhaps medians in the box plot and a Mann Whitney U for comparison would be more accurate. Similarly, if the values in the 2x2 contingency table are small, consider a Fisher's exact test rather than chi-square for the incidence of hypersensitivity reaction comparison (although it likely won't make a significant difference).
- Was there a protocol to say every patient should have a level with every dose, or were levels obtained at random/provider discretion? Could there then be a selection bias in the patients who have activity levels obtained since so many were missed? I'm assuming first dose levels were "missed" because the risk of inactivation with the first dose should theoretically be zero, so they were perhaps more accurately intentionally omitted. 

**Results: **

- Is there any possibility that the level on day 6-8 of 2.8 was spurious? Were there any concomitant levels in this patient with this dose to make sense of such a high level?
- The one patient being described as having silent inactivation - I do not think I would characterize this as silent inactivation technically, since they had detectable activity levels at several time points, and no peak level is ever undetectable. I would consider this a transient accelerated clearance with dose 2 and 3. If silent inactivation was truly present, a patient should not have activity present for a full week and then have full activity with later doses. Patients with activity levels even as low as ~0.02 may still have asparagine depletion (AALL07p4).
- It may be interesting to graph levels over time in some of these patients with very low levels. Was there ever more than one level obtained per dose? Were repeat levels obtained to verify the low activity levels or follow the activity level trend over time (as is suggested in some guidelines?). This would help characterize the PK better in these patients.
- Was the choice to pre-medicate done at a strict, uniform point in time (i.e., was this a quasi-experimental design where a protocol to pre-medicate all patients was mandated?), or was pre-medication at the choice of the treating provider? If this was selected by the provider and not systematic, could there have been some slight selection bias where patients perceived to be at higher risk of hypersensitivity reactions were given pre-medication? This could have diminished the impact of pre-medication on reducing the rate of hypersensitivity reactions. Consider this in the discussion section if deemed relevant.

**Discussion:**

- I would more accurately call the silent inactivation here "accelerated clearance" as the patient had asparagine depletion for at least a week, even with the low level.
- Consider discussing the possible value of obtaining activity levels in patients with questionable allergic reactions (grade 1/2). 
- Could add as a limitation that it appears patients only had one activity level obtained per dose, which makes it challenging to interpret PK, especially in patients with higher or lower than expected activity levels. 

Overall, a very well-done paper with logical conclusions. Thank you for allowing me to review the manuscript and for your hard work in this research.

I confirm that I have read this submission and believe that I have an appropriate level of expertise to confirm that it is of an acceptable scientific standard, however I have significant reservations, as outlined above.

## References

[1] : Universal premedication and therapeutic drug monitoring for asparaginase-based therapy prevents infusion-associated acute adverse events and drug substitutions. *Pediatr Blood Cancer*.2019;66(8) : 10.1002/pbc.27797 e27797 10.1002/pbc.27797 31099154PMC8294186

[2] : A single-center multidisciplinary approach to managing the global Erwinia asparaginase shortage. *Leuk Lymphoma*.2019; 10.1080/10428194.2019.1608530 1-15 10.1080/10428194.2019.1608530 31099289

[3] : Plasma asparaginase activity and asparagine depletion in acute lymphoblastic leukemia patients treated with pegaspargase on Children's Oncology Group AALL07P4. *Leuk Lymphoma*.2019;60(7) : 10.1080/10428194.2018.1542146 1740-1748 10.1080/10428194.2018.1542146 30626253PMC6594900

[4] : A Pediatric-Inspired Regimen Containing Multiple Doses of Intravenous Pegylated Asparaginase Appears Safe and Effective in Newly Diagnosed Adult Patients with Ph-Negative Acute Lymphoblastic Leukemia in Adults up to Age 60: Results of a Multi-Center Phase II Clinical Trial. *Blood*.2016;128(1629) :

# Response

**Published:** 2019-12-19

**Authors:** Bruce Bostrom

**Affiliations:** Children's Hospitals and Clinics of Minnesota, Minneapolis, USA

**Bernard L. Marini**, Department of Pharmacy Services and Clinical Sciences, Michigan Medicine, University of Michigan College of Pharmacy, Ann Arbor, MI, USA 

Drs. Losasso, Bostrom, and Messinger should be applauded for their work in characterizing asparaginase activity levels in ALL patients. The results and research are clearly presented and accurately reported. The conclusions of the study are appropriate, and the limitations of this retrospective cohort study are well-described.

Some specific suggestions/comments/recommendations:

**Abstract:**

- I would include the n in the incidence of hypersensitivity reactions, or acknowledge that the study may not have been powered to assess this outcome. Readers who only look at the abstract may wrongly conclude pre-medication is not effective, when the analysis may have been underpowered to detect a statistically significant difference in hypersensitivity reactions in the pre-medication vs. no pre-medication group.Response: done. Number of patients was added.  Dr Marini is correct that the study was underpowered and we have added statements to that effect in the abstract and first paragraph of the discussion.
- The paper, methods, and title are focused on the impact of pre-medication on activity levels; however, the conclusion primarily talks about the low incidence of silent inactivation with two doses; thus monitoring only being necessary in patients that receive >2 doses. I would consider a sentence re: pre-medication here as well. Response: done. Sentence added: “Though premedication resulted in lower allergic reactions the difference was not statistically different due to small numbers.” **Intro:**

- Not essential but could consider mentioning fibrates in the treatment of hypertriglyceridemia, which is another first line option.Response: done and referenced.
- Would cite more recent data on the impact of pre-medication on preventing hypersensitivity reactions - Cooper SL, Young DJ, Bowen CJ, Arwood NM, Poggi SG, Brown PA. Universal premedication and therapeutic drug monitoring for asparaginase-based therapy prevents infusion-associated acute adverse events and drug substitutions. Pediatr Blood Cancer. 2019 Aug;66(8):e27797. ^1^ and Marini BL, Brown J, Benitez L, Walling E, Hutchinson RJ, Mody R, Jasty Rao R, Slagle L, Bishop L, Pettit K, Bixby DL, Burke PW, Perissinotti AJ. A single-center multidisciplinary approach to managing the global Erwinia asparaginase shortage. Leuk Lymphoma. 2019 May 17:1-15 ^2^. Response: Reference 1 is already there in the addendum and added the reference 2 to the addendum.
- I would be careful with stating asparaginase antibodies are always predictive of future allergic reactions, as not all antibodies inactivate asparaginase or lead to clinical hypersensitivity reactions (ex: CCG01961). Consider rewording to soften this language.Response we now quote exactly the sensitivity and specificity reported by the St. Jude group [Liu et al. Leukemia. 2012 Nov;26(11):2303-9] which addresses your concern.
- In paragraph 5, the statement is made that silent inactivation is of greater concern than allergic reactions. However, one could argue that allergic reactions are more common than silent inactivation with asparaginase (~1%), and monitoring activity levels in the setting of grade 1-2 allergic reactions is essential to determine which patients with clinical hypersensitivity reactions are having true antibody mediated reactions vs. other, non-antibody mediated (possibly ammonia vs. other immune-mediated) allergic reactions that would not warrant a switch to another asparaginase preparation. Response: we altered the paragraph somewhat and specified: “…potentially of greater concern than bone-fide allergic reactions, as patients with grade 3-4 allergic reactions to pegaspargase will be switched to erwinase, which theoretically will improve outcome.”As you suggested at the end of the paragraph we also added this sentence: “Additionally, the low grade 1-2 allergic reactions that are more common than silent inactivation do require pegaspargase activity monitoring to ensure a switch to erwinase if confirmed as true inactivation.”
- Other large scale studies assessing the rate of silent inactivation and finding the rate to be <8% could also be included in this discussion (or cited). These include, AALL07P4, Schore, et al. Leuk Lymph 2019 ^3^, CCG1962, and Park JH, et al. Blood. 2016;128:1629 ^4^.Response: Done. Also quoted Place et al. “Notably, lower silent inactivation with pegaspargase than native Escherichia coli asparaginase have been reported. [15-17]” **Methods:**

- While a sample size was not calculated a priori, could the authors conduct a sensitivity analysis on the data determined the detectable effect size at 80% power in the primary outcome of interest (rate of grade 3/4 allergic reactions?)?Response: A sentence in first paragraph of the discussion was changed as follows: “Compared with historical controls that received similar therapy, premedication did reduce the incidence of grade 3 or 4 allergic reactions when measured per patient or per dose of pegaspargase. The power calculation was only 0.54 per patient and 0.24 per pegasparaginase dose thus our study was underpowered to show statistical significance.”
- Were dose reductions in PEG ever conducted in this data set for toxicity/age/risk factors for hepatotoxicity? Response: No none. All PEG doses were given at a dose of 2500 units/m2, as documented in methods.
- It is unclear how silent inactivation is defined in this data-set from the methods. It may also be clinically relevant to capture "accelerated clearance", which also may be clinically relevant. Not all anti-asparaginase antibodies lead to full inactivation of the drug, but some lead to accelerated clearance and lower than expected values at early time points. A patient with silent inactivation would have a peak level that is undetectable, whereas a patient with accelerated clearance may clear the PEG by day ~7 when an expected duration of asparagine depletion is 21-28 days with a full pediatric dose.  Response: we would suggest that prior studies defined silent inactivation similarly to our study. Here none of the measurements in the current study have included peak levels. Thus, like others we would suggest that accelerated clearance without noted clinical symptoms is the same as silent inactivation.
- Were any levels obtained in patients with low-grade hypersensitivity reactions? If so, did any of these patients have accelerated clearance/"loud" inactivation?Response: As noted – we did not collect low-grade (level 1-2) reactions therefore we cannot answer this question unfortunately.
- Were the activity levels normally distributed? If not, perhaps medians in the box plot and a Mann Whitney U for comparison would be more accurate. Similarly, if the values in the 2x2 contingency table are small, consider a Fisher's exact test rather than chi-square for the incidence of hypersensitivity reaction comparison (although it likely won't make a significant difference).Response: The data was normally distributed by visual inspection of the normal curve and Kolmogorov-Smirnov test of normality, WITH THE EXCEPTION of the day 6-8 pegasparaginase activity level.   As Dr Marini pointed out this contained an extreme outlier which we could not explain. Thus we elected to remove this outlier and the distribution was normal.  The removal of this was discussed in the methods.Data was reanalyzed and graphs redone after this change. Analysis showed a statistically significant difference in the day 6-8 pegaspargase level with and without premeds (lower without premed.  Of note a significant difference was also seen with the dataset containing the outlier.  Also analysis of the day 3-5 and 10-12 pegaspargase levels did not show a significant difference with premeds perhaps due to the small sample sizes.The finding of difference in the pegasparaginase level with premeds has not been previously described to our knowledge. This may be of clinical significance and should, if possible confirmed in other retrospective studies.  Given the increased acceptance of premeds for PEG it is unlikely and perhaps unethical to confirm in a prospective randomized trial, in our opinion.  Text relating to this new finding has been added to the manuscript in various places.
- Was there a protocol to say every patient should have a level with every dose, or were levels obtained at random/provider discretion? Could there then be a selection bias in the patients who have activity levels obtained since so many were missed? I'm assuming first dose levels were "missed" because the risk of inactivation with the first dose should theoretically be zero, so they were perhaps more accurately intentionally omitted. Response: our protocol did instruct that all pegaspargase doses will be followed by levels, as outlined in the methods. Compliance with this instruction was not complete at all as we outlined in the results. We think that there was no collection bias but rather non-compliance. It is true also that there is a misconception that risk of inactivation after the first dose is zero. Please note the recent report from Mary Relling group suggesting that many allergic reactions are actually to the PEG and may occur after the 1 ^st^ dose of pegaspargase. We in fact have seen patients develop anaphylaxis right after the 1 ^st^ dose. (Liu Y, Smith CA, Panetta JC, Yang W, Thompson LE, Counts JP, Molinelli AR, Pei D, Kornegay NM, Crews KR, Swanson H, Cheng C, Karol SE, Evans WE, Inaba H, Pui CH, Jeha S, Relling MV. Antibodies Predict Pegaspargase Allergic Reactions and Failure of Rechallenge. J Clin Oncol. 2019 Aug 10;37(23):2051-2061. doi:10.1200/JCO.18.02439. Epub 2019 Jun 12. PubMed PMID: 31188727.) **Results: **

- Is there any possibility that the level on day 6-8 of 2.8 was spurious? Were there any concomitant levels in this patient with this dose to make sense of such a high level?Response: this patient (UPN 11) after dose 3 of PEG had level of 2.8 on day 7.  The scanned faxed report from AI Biotech was checked and confirmed the result of 2.8 as reported.  We have no other levels after dose 3 to validate this result.  After the preceding dose (#2) the result on day 7 was 0.78 and after the subsequent dose (#)4 the result on day 11 was 0.97.  Based on the extreme deviance from a normal distribution we elected to remove this from the graphs and data analysis as discussed above.
- The one patient being described as having silent inactivation - I do not think I would characterize this as silent inactivation technically, since they had detectable activity levels at several time points, and no peak level is ever undetectable. I would consider this a transient accelerated clearance with dose 2 and 3. If silent inactivation was truly present, a patient should not have activity present for a full week and then have full activity with later doses. Patients with activity levels even as low as ~0.02 may still have asparagine depletion (AALL07p4).Response: It is our suggestion that accelerated clearance occurs through immune mechanism – and as we suggested above is similar to silent inactivation. Though it is possible that the patient had transient accelerated clearance with dose 2 and 3 as an explanation, it remains possible that that patient had transient silent inactivation after dose 3 and had become tolerant PEG when continued dose administered as we explain in the discussion paragraph 2 noting to Tong papers.
- It may be interesting to graph levels over time in some of these patients with very low levels. Was there ever more than one level obtained per dose? Were repeat levels obtained to verify the low activity levels or follow the activity level trend over time (as is suggested in some guidelines?). This would help characterize the PK better in these patients.Response: the patient UPN 28 with the silent activation did have level 0.05 day 8 then 0.00 day 16. It verified the very low levels. No others had confirmatory low levels.
- Was the choice to pre-medicate done at a strict, uniform point in time (i.e., was this a quasi-experimental design where a protocol to pre-medicate all patients was mandated?), or was pre-medication at the choice of the treating provider? If this was selected by the provider and not systematic, could there have been some slight selection bias where patients perceived to be at higher risk of hypersensitivity reactions were given pre-medication? This could have diminished the impact of pre-medication on reducing the rate of hypersensitivity reactions. Consider this in the discussion section if deemed relevant.As we suggested above these were mandated premedications given at a specified time. It was not at the choice of the treating provider; therefore bias seems unlikely. **Discussion:**

- I would more accurately call the silent inactivation here "accelerated clearance" as the patient had asparagine depletion for at least a week, even with the low level.We respectfully disagree as we consider accelerated clearance equal to silent inactivation, since they actually are the same mechanism.
- Consider discussing the possible value of obtaining activity levels in patients with questionable allergic reactions (grade 1/2). Response: Thank you. This was added to the last paragraph of the discussion.
- Could add as a limitation that it appears patients only had one activity level obtained per dose, which makes it challenging to interpret PK, especially in patients with higher or lower than expected activity levels. Response: Thank you, added to the limitation paragraph.Overall, a very well-done paper with logical conclusions. Thank you for allowing me to review the manuscript and for your hard work in this research.

- **Is the work clearly and accurately presented and does it cite the current literature?**Yes
- **Is the study design appropriate and is the work technically sound?**Partly
- **Are sufficient details of methods and analysis provided to allow replication by others?**Yes
- **If applicable, is the statistical analysis and its interpretation appropriate?**Partly
- **Are all the source data underlying the results available to ensure full reproducibility?**Yes
- **Are the conclusions drawn adequately supported by the results?**Yes

**References**

1. Cooper SL, Young DJ, Bowen CJ, Arwood NM, Poggi SG, Brown PA: Universal premedication and therapeutic drug monitoring for asparaginase-based therapy prevents infusion-associated acute adverse events and drug substitutions. *Pediatr Blood Cancer*. 2019; **66** (8): e27797 PubMed Abstract | Publisher Full Text

2. Marini BL, Brown J, Benitez L, Walling E, Hutchinson RJ, Mody R, Jasty Rao R, Slagle L, Bishop L, Pettit K, Bixby DL, Burke PW, Perissinotti AJ: A single-center multidisciplinary approach to managing the global Erwinia asparaginase shortage. *Leuk Lymphoma*. 2019. 1-15 PubMed Abstract | Publisher Full Text

3. Schore RJ, Devidas M, Bleyer A, Reaman GH, Winick N, Loh ML, Raetz EA, Carroll WL, Hunger SP, Angiolillo AL: Plasma asparaginase activity and asparagine depletion in acute lymphoblastic leukemia patients treated with pegaspargase on Children's Oncology Group AALL07P4. *Leuk Lymphoma*. 2019; **60** (7): 1740-1748 PubMed Abstract | Publisher Full Text

4. Park JH, Ritchie EK, Rao AV, Devlin S, Hsu M, Khawaja TT, O’Donnell B, Cazacu MI, Stopka DM, Tallman MS, Douer D: A Pediatric-Inspired Regimen Containing Multiple Doses of Intravenous Pegylated Asparaginase Appears Safe and Effective in Newly Diagnosed Adult Patients with Ph-Negative Acute Lymphoblastic Leukemia in Adults up to Age 60: Results of a Multi-Center Phase II Clinical Trial. *Blood*. 2016; **128** (1629).
